# Identification of a Novel Linear B Cell Epitope on the Sao Protein of *Streptococcus suis* Serotype 2

**DOI:** 10.3389/fimmu.2020.01492

**Published:** 2020-07-16

**Authors:** Jing Wang, Ruirui Dong, Ping Zou, Yuejuan Chen, Na Li, Yao Wang, Ting Zhang, Xiuzhen Pan

**Affiliations:** ^1^The Affiliated Wuxi Maternity and Child Health Care Hospital of Nanjing Medical University, Wuxi, China; ^2^Department of Microbiology, Hua Dong Research Institute for Medicine and Biotechnics, Nanjing, China

**Keywords:** *Streptococcus suis*, surface antigen one, B-cell epitope, ELISA, synthetic peptide, homology analysis

## Abstract

Surface antigen one (Sao) protein is a bacterial surface protein identified in the important zoonotic pathogen *Streptococcus suis* serotype 2 (*S*. *suis 2*) during an extensive search for functional proteins. The Sao protein is anchored to the bacterial cell wall by the LPVTG motif and is widely distributed in many *S*. *suis* serotypes. In this paper, we present the immunodominant epitope peptide of the Sao protein that is recognized by BALB/c antibodies against the Sao protein: ^355^SEKQMPSVVNENAVTPEKQMTNKENDNIET^384^ (location Sao_355−384_). To determine the core epitope recognized by antibodies, we prepared truncation peptide libraries. Analyses of the immunoreactivity of truncation peptides with anti-Sao_355−384_ serum revealed that the most immunoreactive sequence was ^355^SEKQMPSVVNENAVTPEK^372^ (location Sao_355−372_). Moreover, we observed that this core epitope also showed good specificity based on the ratio of reactivity with serum from *S*. *suis*–positive patients compared to serum from *S*. *suis*–negative patients. Our results point to the potential of using the Sao_355−372_ peptide in diagnostic assays to determine *S*. *suis* infection in humans.

## Introduction

*S*. *suis* is an important swine pathogen that can be transmitted to humans through contact with diseased animals or contaminated raw pork products (1). This pathogen may induce the overproduction of proinflammatory cytokines, which may lead to septic shock and the activation of different leukocyte populations, thus causing acute inflammation of the central nervous system (CNS). *S*. *suis* can also activate microglia and astrocytes to cause acute inflammatory reactions in the brain, leading to brain edema, cerebrovascular injury, deafness, and other serious intracranial complications (2). In fact, *S. suis* has been reported in many regions, such as the United States, Western Europe, Canada, Australia, Japan, New Zealand, and Southeast Asian (1, 2), in particular China. In the 2005 outbreak in the Chinese province of Sichuan, a significant proportion of patients infected with *S*. *suis* experienced streptococcal toxic shock syndrome (STSS) with high mortality (3). *S*. *suis* undoubtedly poses a threat to public health. Therefore, establishing an ELISA for diagnosing *S. suis* infection is important for epidemiological surveillance.

Although there are many methods of identifying *S*. *suis*, they have several limitations. Conventional bacterial culture, the standard method of identifying *S*. *suis*, usually required accurate control of the sampling time (such as before using antibacterial drugs) and strict sterility. Polymerase chain reaction (PCR) (4, 5) and loop-mediated isothermal amplification (LAMP) assays (6) designed to amplify specific *S*. *suis* genes are also capable of detecting *S*. *suis*. However, these molecular techniques require technical expertise and expensive equipment and therefore are not suitable for use in small hospitals or in the field. The use of serological techniques remains the basic method of diagnosing this disease.

The Sao protein was discovered in 2006 during research on the pathogenesis and functional proteins of *S*. *suis 2*. Sao is a surface protein of *S*. *suis* that is widely present in various serotypes (7). The three allelic variants of the *sao* gene (*sao-s, sao-m*, and *sao-l*) have different lengths. These differences are due to heterogeneity within the number of C-terminal repeat sequences. Sao-m is the most prevalent variant, comprising about 80% of cases, followed by sao-l. Sao-s is detected in no more than 6% of cases. In our previous studies, we showed that the Sao protein is a highly immunoreactive protein that can be used as a marker of *S*. *suis* infection (8). The only disadvantage is that the stability of recombinant Sao protein is poor, which makes it difficult to purify. These factors hinder its development as a marker for detecting *S*. *suis*.

Synthetic peptides have a definite chemical composition, high purity, and high stability and preservability. Short synthetic peptides may replace protein antigen epitopes to capture the specific antibodies from serum samples. Synthetic peptide-based diagnosis can be efficient and accessible in immunodiagnostics. Several studies have investigated the use of synthetic peptides in diagnosing bacterial (9, 10), viral (11–14), and parasitic (15–17) infections in humans, and animals. However, there have been no reports on the use of synthetic peptides to detect *S*. *suis* infection. In this study, we determined the most specific epitope of Sao and prepared synthetic peptides for evaluation as markers of *S*. *suis* infection in enzyme-linked immunosorbent assay (ELISA) using human *S*. *suis*–positive and *S*. *suis*–negative serum.

## Materials and Methods

### Animals, Antigens, and Serum

Six- to 8-weeks-old SPF female BALB/c mice were purchased from Shanghai Laboratory Animal Center, Chinese Academy of Sciences (Shanghai, China). Recombinant Sao-M (rSao-M) was expressed in *E*. *coli* BL21, which was established previously (2). Convalescent serum of 11 patients infected with *S. suis 2* were collected. Convalescent serum of patients infected with *Klebsiella pneumoniae, Escherichia coli, Staphylococcus epidermidis*, and *Enterococcus faecium* were collected at 7–14 days after infection.

### Sequence Characteristics of Sao-M Protein

**Figure 1:**
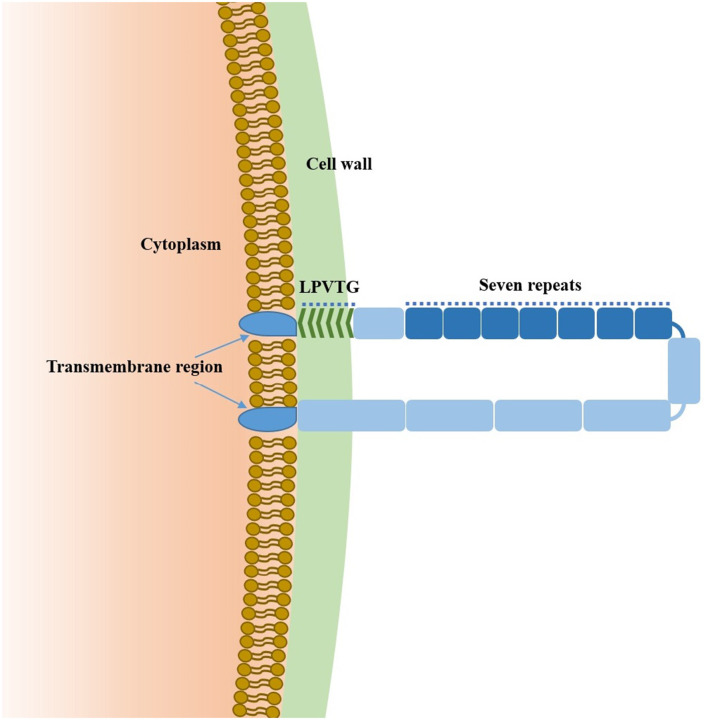
Model of Sao-M protein from *S*. *suis 2*.

Sao-M protein consists of 580 amino acids and is anchored to the cell wall via a C-terminal LPVTG motif. The TMHMM Server (TMHMM Server, RRID: SCR_014935) predicted two transmembrane regions of the Sao protein (7–29 aa, 557–574 aa) and predicted that the intermediate sequences (30–556 aa) were extracellular. Moreover, 295–504 aa were highly repetitive regions of the Sao-M protein, and each region was composed of 30 aa, with seven regions in total (Figure 1).

### Prediction of Immunoinformatics Parameters and Linear B Cell Epitopes of Sao-M Protein

IEDB's Parker hydrophilicity prediction (Immune Epitope Database and Analysis Resource (IEDB), RRID: SCR_006604), Karplus and Schulz flexibility prediction, Emini surface accessibility prediction, and Chou and Fasman beta-turn prediction tools were used to predict the hydrophilicity, flexibility, surface accessibility, and β-sheet of the Sao-M protein, respectively. Three types of bioinformatics software were used to simultaneously predict the linear B cell epitope of Sao-M protein: Bepipred (http://www.cbs.dtu.dk/services/Bepipred/), COBEpro (http://scratch.proteomics.ics.uci.edu/), and ABCpred (http://www.imtech.res.in/raghava/abcpred/). By comprehensively analyzing the prediction results of B cell epitopes and immunoinformatics parameters, we were able to select a linear B cell epitope peptide of Sao-M protein.

### ELISA for Screening Immunodominant Linear B Cell Epitope Peptides

To determine the immunodominant linear B cell epitope peptides of Sao-M, we collected serum samples from BALB/c mice immunized with rSao-M plus Freund's adjuvant. Six- to 8-weeks-old SPF female BALB/c mice were injected once subcutaneously with 100 μg rSao-M protein plus an identical volume of complete Freund's adjuvant (CFA) on day 0. Two subcutaneous booster injections of the same volume with incomplete Freund's adjuvant (IFA) were administered on days 14 and 28. Seven days after the last immunization, anti-rSao-M serum was collected for analyses.

Indirect ELISA was used to detect antibody titer of anti-rSao-M serum. ELISA plate (96 well EIA/RIA plate, Corning Incorporated, United States) was coated with rSao-M protein (100 ng/well) and BSA (negative control protein) in coating buffer (Solarbio) overnight at 4°C. Then wells were blocked with 3.0% BSA in phosphate-buffered saline (PBS). After the wells were washed, anti-rSao-M serum of different dilutions (1:100, 1:200, 1:400, 1:800, 1:1600, 1:3200, 1:6400, 1:12800, 1:25600, 1:51200, 1:102400,1:204800,1:409600, diluted with PBS containing 1% BSA) were incubated in the wells for 1 h at 37°C. After washing away all unbound material, peroxidase-conjugated goat anti-mouse IgG (Bioss, 0.5 μg/ml) was added for 1 h. After being washed, plate was incubated with a TMB substrate (Beyotime) at 37°C for 10 min. We stopped the enzymatic reactions by adding termination fluid (Solarbio), and endpoint absorbance (A) was read at 450 nm. Recorded the highest serum dilution which significantly higher than the negative control.

Indirect ELISA was also used to screen immunodominant linear B cell epitope peptides. ELISA plate was coated with 10 candidate B cell epitope peptides (100 ng/well), GST_141−158_ (irrelevant negative control peptide), rSao-M protein (positive control, 100 ng/well), and BSA (negative control protein) in coating buffer overnight at 4°C. Then wells were blocked with 3.0% BSA in PBS. After the wells were washed, anti-rSao-M serum of four different dilutions (1:1000, 1:2000, 1:4000, 1:8000, diluted with PBS containing 1% BSA) were incubated in the wells for 20 min at 37°C. After any unbound material was washed off, peroxidase-conjugated goat anti-mouse IgG (Bioss, 0.5 μg/ml) was added for 1 h. The next ELISA steps were the same as described above.

### Preparation of Antiserum Against Peptides of Immunodominant B Cell Epitope

To identify the core epitopes of the immunodominant epitope peptide, we produced anti-immunodominant peptide sera in BALB/c mice with one subcutaneous injection and two booster injections of 100 μg KLH-conjugated peptide plus an identical volume of Freund's adjuvant on days 0, 14, and 28. Serum was collected 7 days after the final booster injection. The titer of anti-immunodominant peptide serum was detected by ELISA. ELISA plate was coated with immunodominant peptide (100 ng/well). Anti-immunodominant peptide sera and negative BALB/c serum of different dilutions (1:3000, 1:9000, 1:27000, 1:81000, 1:243000, 1:729000, diluted with PBS containing 1% BSA) were incubated. Other ELISA steps were the same as described above. Recorded the highest serum dilution which significantly higher than the negative control.

### Screening for Core Epitopes Corresponding to the Immunodominant Epitope Peptide

**Figure 2:**
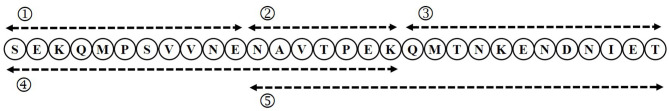
Schematic of truncated peptides.

To identify the core epitopes of the immunodominant epitope peptide, we designed different truncated peptides that could cover its full length (Figure 2). These truncated peptides were synthesized by Jill Biochemical (Shanghai, China). ELISA plate was coated with these five truncated peptides (50, 100, 200, 400 ng/well, respectively), irrelevant negative control peptide (GST_141−158_), positive control peptide Sao_355−384_, negative control protein (BSA), positive control rSao-M protein, and in coating buffer (Solarbio) overnight at 4°C. Then wells were blocked with 3.0% BSA in PBS. After the wells were washed, anti-immunodominant peptide serum of four different dilutions (1:1000,1:2000,1:4000,1:8000, diluted with PBS containing 1% BSA) were incubated in the wells for 1 h at 37°C. The next ELISA steps were the same as described above.

### Preliminary Application of the Core Epitope in Detecting *S*. *suis 2* Infection

The ELISA method was used for preliminary analyses of whether the core epitope had the ability to detect *S*. *suis 2* infection. ELISA plate was coated with the core epitope (100 ng/well) in coating buffer (Solarbio) overnight at 4°C. After the wells were blocked with 3.0% BSA and washed, human serum from different sources of four dilutions (1: 200, 1: 400, 1: 800, 1: 1600, diluted with PBS containing 1% BSA) were added and the ELISA plate was incubated at 37°C for 20 min. After all unbound material was washed off, a peroxidase-conjugated goat anti-human IgG (H+L; Biodragon) was added for 1 h. The next ELISA steps were the same as described above.

### Sequence Homology

To determine whether the core epitope was conserved among *S*. *suis* strains, we analyzed the Sao protein amino acid sequences of 17 *S*. *suis* strains with DNASTAR (GenVision, RRID: SCR_001166). Sao proteins from 17 serotypes of *S*. *suis* had previously been sequenced by the Hua Dong Research Institute for Medicine and Biotechnics. A heatmap for homology comparison between Sao_355−372_ in *S*. *Suis 2* and the 17 *S*. *Suis* strains was created with TBTools (http://www.tbtools.com/).

### Localization of the Core Epitope on Sao

SWISS-MODEL (SWISS-MODEL, RRID: SCR_018123) was used to predict the three-dimensional structure of the SAO-M protein. Two threading templates were selected by this program for construction (4s3l.1, 4gjp.1). Figures were generated with the SWISS-MODEL visualization system. Core epitopes were mapped against the three-dimensional structure of the SAO-M protein.

### Statistical Analysis

Analyses were performed using an unpaired Student's *t*-test in the software GraphPad Prism 5.0: *P* < 0.05 was considered statistically significant.

## Results

### Ten Linear B Cell Epitopes Screened by Prediction

**Figure 3:**
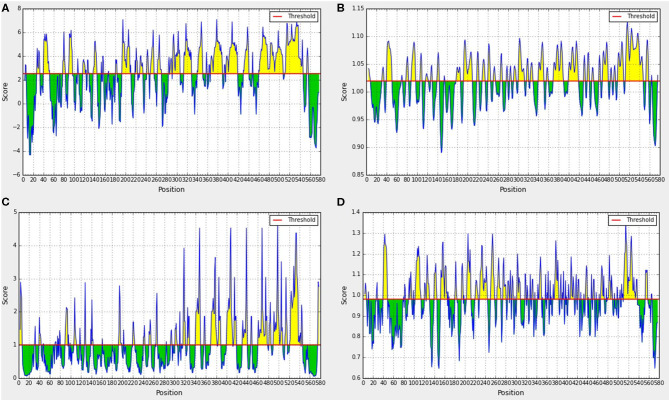
Prediction results of immunoinformatics parameters of Sao-M protein. **(A)** Hydrophilicity analyses, **(B)** Flexibility analyses, **(C)** Surface accessibility analyses, **(D)** β-sheet analyses.

IEDB bioinformatics analysis tools were used to predict the hydrophilicity, flexibility, surface accessibility, and β-sheet of the Sao-M protein. Yellow areas above the set threshold in Figure 3 are possible hydrophilic (54.36%; Figure 3A), flexible (52.18%; Figure 3B), surface accessibility (37.46%; Figure 3C), and β-fold (50.70%; Figure 3D) areas. The results of the three linear B cell epitope prediction tools were summarized and compared. The overlapping regions were combined with the immunoinformatics parameters to predict the results. Finally, 10 linear B cell epitope peptides were screened. Epitope peptides were synthesized with the assistance of Jill Biochemical. The basic sequences were as shown in Table 1.

**Table 1:** Amino acid sequence of 10 candidate B-cell epitopes of Sao-M protein.

**SEQ ID**	**Location**	**Purity (%)**	**Sequence (N → C)**
NO. 1	Sao_37−47_	92.30	ISAKQPDGGQA
NO. 2	Sao_92−98_	90.37	NTKEGQV
NO. 3	Sao_106−111_	98.28	DKPGKY
NO. 4	Sao_149−164_	93.22	IEFIDNDNVFNNFYTP
NO. 5	Sao_205−212_	91.79	QADGSVNF
NO. 6	Sao_217−223_	91.74	FTKEGTY
NO. 7	Sao_278−293_	93.75	GKLIAPTTDSVITDNE
NO. 8	Sao_355−384_	94.92	SEKQMPSVVNENAVTPEKQMTNKENDNIET
NO. 9	Sao_396−402_	97.20	NAVTPEK
NO. 10	Sao_525−531_	92.43	NTKPTTE
NO. 11	GST_141−158_	96.86	TYLNGDHVTHPDFMLYDA

### Identification of the Immunodominant Epitope Peptide of the Sao-M Protein

**Figure 4:**
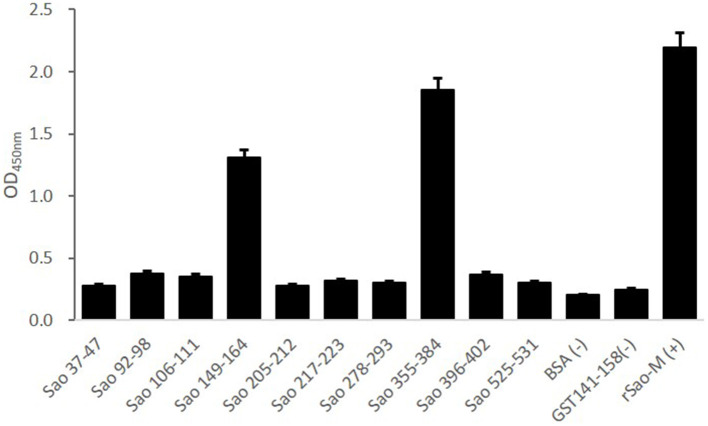
ELISA detection of B cell epitope peptides of Sao-M. To determine the immunodominant epitope peptide of Sao-M, ELISA plate was coated with 10 synthetic peptides or GST_141−158_ (negative control of irrelevant peptide) or rSao-M protein and BSA. Then, serum samples from BALB/c mice that were immunized with rSao-M plus Freund's adjuvant were detected. The absorbance was read at 450 nm. The absorbances at 450 nm for the peptides Sao_355−384_ and Sao_149−164_ were significantly higher than GST_141−158_ (*P* < 0.0001) and BSA (*P* < 0.0001). Of these two immunodominant epitope peptides, Sao_355−384_ had the stronger response.

Ten B cell epitope peptides of the Sao-M protein of *S*. *suis 2* were synthesized. To determine the immunodominant peptides of Sao-M, we collected anti-rSao-M serum samples from BALB/c mice that had been immunized with rSao-M plus Freund's adjuvant. The titer of the anti-rSao-M serum prepared in this study was >1:400,000, which is suitable for identifying immunodominant linear B cell epitope peptides. All 10 B cell epitope peptides were scanned with ELISA. The optimal dilution of anti-rSao-M serum was 1:1000 (Table 2). The ELISA results indicated that the strongest IgG antibody reactivity was concentrated on two major immunodominant peptides: Sao_355−384_ (SEKQMPSVVNENAVTPEKQMTNKENDNIET) and Sao_149−164_ of the Sao-M protein (*P* < 0.0001; Figure 4). Of these two immunodominant epitope peptides, Sao_355−384_ had the stronger response.

**Table 2:** Determination of the coating amount of antigen and the working concentration of serum.

**Anti-rSao-M serum dilution**	**Coated amount of Sao**_**355−384**_ **(ng/well) sequence (N → C)**				**GST_**141−158**_ (ng/well)**
	**50**	**100**	**200**	**400**	**200**
1:1000	2.746	**3.122**	2.799	3.071	0.956
1:2000	2.531	2.642	2.716	2.853	0.739
1:4000	2.087	2.135	2.309	2.420	0.646
1:8000	1.523	1.706	1.948	2.024	0.425
**Anti-Sao**_**355−384**_ **serum dilution**	**Coated amount of Sao**_**355−372**_ **(ng/well) sequence (N → C)**				**GST**_**141−158**_ **(ng/well)**
	**50**	**100**	**200**	**400**	**200**
1:1000	2.096	2.780	**2.844**	2.835	0.889
1:2000	1.907	2.284	2.588	2.782	0.781
1:4000	1.387	1.683	2.106	2.221	0.616
1:8000	0.742	1.104	1.575	1.588	0.272
**Convalescent serum dilution**	**Coated amount of Sao**_**355−372**_ **(ng/well) sequence (N → C)**				**GST**_**141−158**_ **(ng/well)**
	**50**	**100**	**200**	**400**	**200**
1:200	2.045	2.213	2.105	2.137	0.874
1:400	2.281	**2.287**	2.204	2.034	0.739
1:800	2.145	2.071	1.816	2.016	0.708
1:1600	1.726	1.704	1.509	1.458	0.412

*The absorbance values for optimal coating amount and serum dilutionun are marked by bold values*.

### Identification of the Core Epitope of the Immunodominant Epitope Peptide

**Figure 5:**
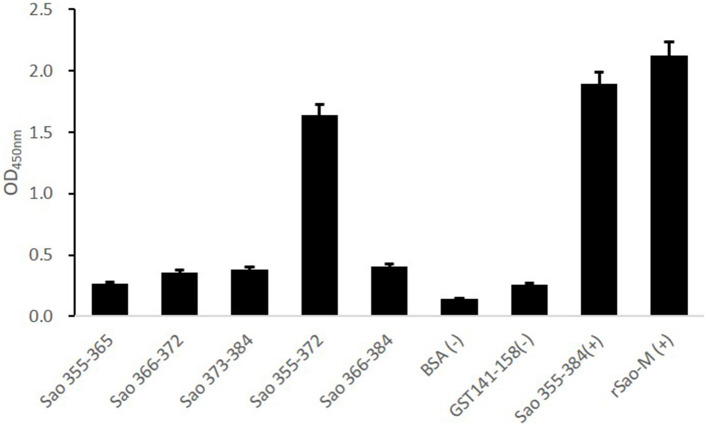
ELISA detection of the core epitope. To determine the core epitope of immunodominant epitope peptide Sao_355−384_, ELISA plate was coated with five truncated peptides or GST_141−158_ (negative control of irrelevant peptide) or Sao_355−384_ (positive control of synthetic peptide) or rSao-M protein (positive control of protein) and BSA. Then, serum samples from BALB/c mice that were immunized with Sao_355−384_ plus Freund's adjuvant were detected. The absorbance at 450 nm for the peptide Sao_355−372_ was significantly higher than GST_141−158_ (*P* < 0.01) and BSA (*P* < 0.01).

To further confirm the core sequences of the immunodominant peptide Sao_355−384_, we designed and synthesized five truncated peptides. The basic sequences are listed in Table 3. Anti-Sao_355−384_ serum samples were collected from BALB/c mice with titer higher than 1:700,000 for use in identifying the core epitope. Truncated peptides with stronger responses to the anti-Sao_355−384_ serum samples were once again scanned with ELISA. The optimal working concentration was 200 ng/well of antigen coating and 1: 1000 anti-Sao_355−384_ serum dilution (Table 2). The ELISA results showed that the strongest response to the anti-Sao_355−384_ serum samples was from Sao_355−372_ (*P* < 0.05; Figure 5).

**Table 3:** Amino acid sequence of five candidate B-cell epitopes of Sao-M protein.

**SEQ ID**	**Location**	**Purity (%)**	**Sequence (N → C)**
NO. 1	Sao_355−365_	98.28%	SEKQMPSVVNE
NO. 2	Sao_366−372_	93.22%	NAVTPEK
NO. 3	Sao_373−384_	91.79%	QMTNKENDNIET
NO. 4	Sao_355−372_	91.74%	SEKQMPSVVNENAVTPEK
NO. 5	Sao_366−384_	93.75%	NAVTPEKQMTNKENDNIET

### Preliminary Application of the Core Epitope in Detecting *S*. *suis 2* Infection

**Figure 6:**
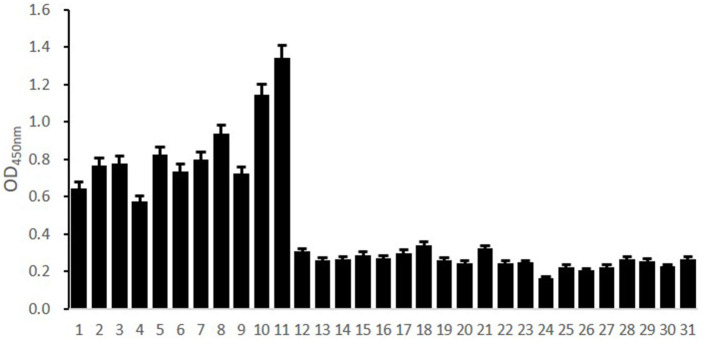
Evaluation of the detection ability of Sao_355−372_ by ELISA. ELISA plate was coated with Sao_355−372_. Then, human serum samples of known origin were detected. Columns 1–11 showed the results of reactions between Sao_355−372_ and antibodies from patients with *S. suis* infection. Columns 12–27 showed the results of reactions between Sao_355−372_ and serum from healthy people. Columns 28–31 showed the results of reactions between Sao_355−372_ and antibodies from patients infected by *Staphylococcus epidermidis, Enterococcus faecium, Klebsiella pneumoniae*, and *Escherichia coli*. The results showed that the core epitope Sao_355−372_ only reacted with antibodies from the 11 patients with *S*. *suis* infection. It did not react with convalescent serum of patients infected with *Klebsiella pneumoniae, Escherichia coli, Staphylococcus epidermidis*, or *Enterococcus faecium*, or with negative serum from healthy people (*P* < 0.05).

To preliminarily evaluate the ability of the core epitope to detect *S*. *suis 2* infection, we used Sao_355−372_ as a detection antigen. The ability of Sao_355−372_ to detect *S*. *suis 2* infection was determined by the antigen–antibody reaction between Sao_355−372_ and human serum of known origin. Human serum of known origin was divided into three groups: one from humans infected with *S*. *suis 2* (11 samples); one from those infected with *Klebsiella pneumoniae, Escherichia coli, Staphylococcus epidermidis*, or *Enterococcus faecium* (four samples); and one from healthy people (16 samples). The optimal working concentration was 100 ng/well of Sao_355−372_ coating and 1: 400 convalescent serum dilution of patients infected with *S.suis 2* (Table 2). The ELISA results showed that the core epitope Sao_355−372_ only reacted with antibodies in the 11 patients with *S*. *suis* infection. It did not react with convalescent serum of patients infected with *Klebsiella pneumoniae, Escherichia coli, Staphylococcus epidermidis*, or *Enterococcus faecium*, or with negative serum from healthy people (*P* < 0.05; Figure 6).

### Homology Analyses

**Figure 7:**
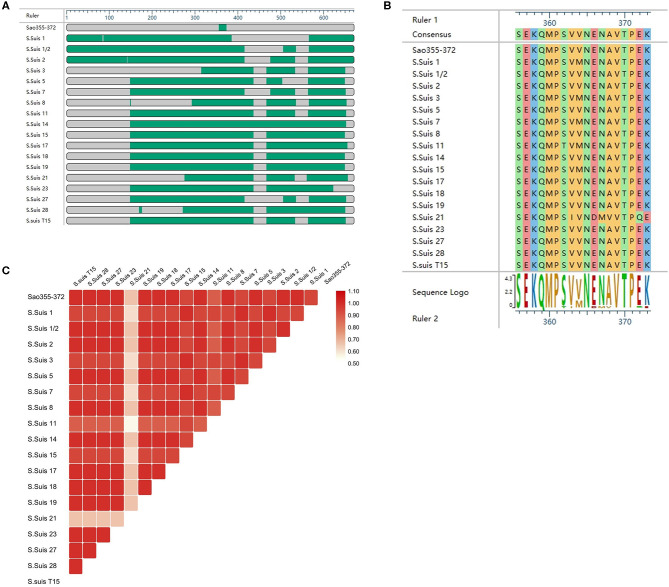
Comparison of the Sao_355−372_ epitope amino acid sequence among different *S*. *Suis* strains. **(A)** Overview of the comparison results. **(B)** Amino acid sequence diagram of the alignment result. **(C)** Heatmap comparing homology between Sao_355−372_ and 17 other *S*. *Suis* strains. Homology was analyzed by DNASTAR and TBTools.

To explore the conservation of the core epitope, we selected the amino acid sequences of Sao_355−372_ of Sao from *S*. *Suis 2* and Sao proteins from 17 *S*. *Suis* isolates for alignment, which was performed with DNASTAR. Sao proteins from the 17 *S*. *Suis* strains had been previously sequenced by the Hua Dong Research Institute for Medicine and Biotechnics. The sequence of the core epitope Sao_355−372_ (SEKQMPSVVNENAVTPEK) was highly conserved among the *S*. *Suis* strains analyzed (Figure 7A). *S*. *Suis 1/2, S*. *Suis 5, S*. *Suis 8, S*. *Suis 14, S*. *Suis 17, S*. *Suis 18, S*. *Suis 19, S*. *Suis 23, S*. *Suis 27, S*. *Suis 28*, and *S*. *Suis T15* shared 100% sequence similarity in the position of epitope Sao_355−372_. *S*. *Suis 1, S*. *Suis 3, S*. *Suis 7*, and *S*. *Suis 15* shared 94.1% sequence similarity with Sao_355−372_, with one amino acid difference in the ninth position (methionine substituted for valine). The amino acid identity was 64.7% for *S*. *Suis 21* (Figure 7B). A heatmap comparing the homology between Sao_355−372_ and Sao proteins from 17 *S*. *Suis* strains was prepared with TBTools (Figure 7C). The heatmap results showed that, with the exception of *S*. *Suis 21*, segments of the Sao protein in the 17 *S*. *Suis* strains were similar in color to the corresponding segments of Sao_355−372_, which indicated that they had good homology.

### Localization of Sao_355−372_ on the Sao Protein

**Figure 8:**
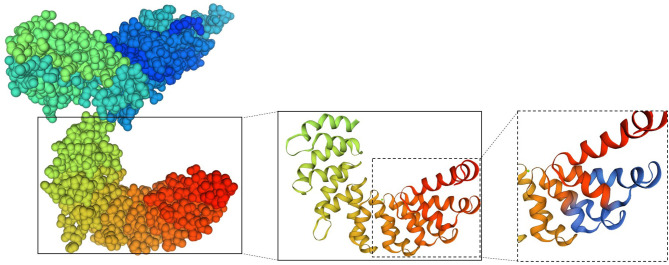
Model of the 3D structure of the Sao-M protein predicted by SWISS-MODEL. The overall structure is shown on the left; the two core epitopes (aa SEKQMPSVVNENAVTPEK) at different positions are marked on the right in blue.

A three-dimensional model of the Sao-M protein was constructed based on templates on the SWISS-MODEL server website (Figure 8). Two monomer templates, 4s3l.1 and 4gjp.1, were selected for construction based on a score that combined the highest sequence coverage and sequence similarity. Sao_355−372_ was predicted in the alpha helix region exposed on the surface of the Sao-M protein, which suggested that the core epitope was very likely a linear epitope. It is worth noting that a segment of the same amino acid sequence as Sao_355−372_ was in the 385–402 position of the Sao-M protein, which was also located in the alpha helix region in close proximity to Sao_355−372_ in the spatial conformation. Two core epitopes at different positions are marked in blue in Figure 8.

## Discussion

*S. suis* infection is the main reason for the sudden death of pigs. Due to the zoonotic capabilities, *S. suis* has increasingly become a threat to human health. Attempts to establish an ELISA for epidemiological testing of *S*. *suis* infection are hampered by the lack of simple and effective detection methods. The strategy and prerequisite for developing effective detection is the identification and characterization of cell surface targets.

Sao is one of the most abundant and conserved *S*. *Suis* proteins. Immunoelectron microscopy has confirmed that the Sao protein is expressed homogeneously and intensively on the cellular surface (7). Immunization with rSao protein elicited a significant humoral antibody response in piglets, and convalescent-phase swine sera contain high titers of antibody against this protein, which suggests that Sao is a potent antigen expressed during *S*. *suis* infection. Therefore, the Sao protein may be an excellent marker for detecting *S*. *suis* infection. However, the proportion of random coils in the protein is as high as 74.21%. Among the secondary structural components of proteins, α-helix and β-sheet chemical bonds have high bond energy; they help maintain the high-level structure and serve as a backbone for the protein. Random coils are irregular peptide chain conformations that are relatively loose, are prone to distortion, and hover on the protein surface. The high proportion of random coils in the Sao protein deters it from forming stable structures. This problem has also been confirmed in practical work. The Sao protein is easily degraded during the purification process, but this degradation can be avoided to some extent by carrying out purification at 16°C or in an ice bath. To maintain stability, the purified Sao protein requires the addition of four protease inhibitors (PMSF, pepsin peptide, leucine peptide, and aprotinin). Even so, the purified Sao protein is stable for only 1 week at 4°C and for 6 months at −20°C. Its susceptibility to degradation hinders its further development as a marker for detecting *S. suis*.

Researchers in many fields have used synthetic peptides derived from immunological determinants to specifically capture cognate antibodies, including bacteria and viruses. When synthetic peptides are used to replace bacterial surface proteins to detect antibodies, they have the advantages of being stable and allowing for easy preservation. The synthetic peptides in the current study are all synthesized by professional biotechnology companies, and the synthesis cycle is short and flexible. The purities of the synthetic peptides are >90%, and they can be stably stored at −20°C for several years in powder form. Therefore, searching for the epitope of the Sao protein as a marker for detecting *S. suis* infection has important clinical value for establishing an ELISA for diagnosing *S*. *suis* infection.

Sao-M (580 aa) of the *S*. *suis 2* (05ZYH33) strain was the focus of the present study. Database resources and bioinformatics technology were used to carry out a comprehensive analysis of the Sao protein. The immunodominant epitope peptide Sao_355−384_ (30aa), which reacts strongly with anti-rSao-M serum, was screened from the predicted 10 linear B cell epitope antigens. Epitopes are the smallest units of immune function in antigens. To further confirm the core sequences, we designed and synthesized five truncated peptides. Anti-Sao_355−384_ serum successfully captured an 18 aa sequence (Sao_355−372_) from the five truncated peptides. We believe that Sao_355−372_ is the core epitope of the Sao protein and can replace Sao as a marker for detecting *S*. *suis* infection. To confirm this, we collected 31 serum samples: 11 samples from patients diagnosed with *S*. *suis* infection, four samples from patients infected with clinically common pathogenic bacteria, and 16 samples of healthy serum. The results showed that Sao_355−372_ reacted strongly only with the serum from patients with *S*. *suis* infection. This result provides a powerful basis for the further development of tools for epidemiological surveillance of *S. suis* infection. Since no evident correlation was found between human convalescent serum dilution and peptide coating antigen (Table 2), the ELISA test requires further optimization. Nevertheless, a clear difference was observed between these sera and control sera.

*S*. *suis* strains are classified into serotypes primarily on the basis of serological reactions against the capsular polysaccharide (CPS). Until relatively recently, 29 serotypes (1–19, 21, 23–25, 27–31, and 1/2) were recognized (1, 18, 19). Among them, 20 serotypes can detect the *sao* gene (1–5, 7, 8, 11, 14, 15, 17–19, 21, 23, 27–30, and 1/2). This shows that Sao is common among different serotypes of *S*. *suis*. To explore the conservation of Sao_355−372_ (from *S*. *suis 2*), we compared it with Sao proteins of different *S*. *Suis* serotypes. The results showed that Sao_355−372_ had a high degree of homology with 15 serotypes (88.2%), which included the main pathogenic *S*. *suis* serotypes: types 1, 1/2, 2, 7, and 14. The amino acid identity with *S*. *Suis 21* was only 64.7%. At present, scholars have performed relatively little research on *S*. *Suis 21*. The literature indicates that *S*. *Suis 21* is a rare bacterium associated with a commensal pathotype composed of isolates from farms with no known history of *S*. *suis*–associated disease (20). Therefore, the low homology of Sao_355−372_ with *S*. *Suis 21* has little bearing on its suitability as a marker for detecting *S*. *Suis* infection.

Furthermore, the location of the Sao_355−372_ epitope was mapped for the first time with three-dimensional homology modeling, which showed that epitope Sao_355−372_ was in the alpha helix region on the surface of the Sao-M protein. An unexpected finding is that a segment of the same amino acid sequence as Sao_355−372_ was in the 385–402 position of Sao-M, which was located in close proximity to Sao_355−372_ in the spatial conformation. This indicates that two effective epitopes were exposed on the surface of the Sao protein, which may indirectly enhance the efficacy of Sao_355−372_ as a detection marker.

In conclusion, our results show that short, synthetic, specific epitopes that belong to the Sao protein can be used to identify the presence of specific antibodies against *S*. *suis* infection in humans. The advantages of these epitopes include their high specificity, high yield, and rapid production process. The presence of two core epitopes on the surface of the same protein could further improve diagnostic tests based on ELISA. Epitope-based diagnostic methods may therefore be established to exploit the utility of antigens for *S*. *suis* detection. However, we strongly emphasize that further research should be performed to augment and refine the existing data.

## Data Availability Statement

The datasets [Sao-M protein sequence] for this study can be found in the [GenBank] [AEG67301.1].

## Ethics Statement

The studies involving human participants were reviewed and approved by Ethics Committee of Wuxi Maternity and Child Health Care Hospital. Written informed consent for participation was not required for this study in accordance with the national legislation and the institutional requirements. The animal study was reviewed and approved by Scientific Research Committee of Laboratory Animal Welfare, Nanjing Medical University.

## Author Contributions

JW designed, coordinated, conceived of the study, and drafted the manuscript. RD performed the peptide synthesis. PZ performed the ELISA. YC performed the preparation of BALB/c polyclonal antibody. NL collected human sera. YW performed the purification. TZ performed homology analyses. XP was a supervisor and helped to draft the manuscript. All authors read and approved the final manuscript.

## Conflict of Interest

The authors declare that the research was conducted in the absence of any commercial or financial relationships that could be construed as a potential conflict of interest.

## References

[1] Segura M Fittipaldi N Calzas C Gottschalk M. Critical *Streptococcus suis* virulence factors: are they all really critical? Trends Microbiol. (2017) 25:585–99. 10.1016/j.tim.2017.02.005 28274524

[2] Dutkiewicz J Zaja̧c V Sroka J Wasiński B Cisak E Sawczyn A Streptococcus suis: a re-emerging pathogen associated with occupational exposure to pigs or pork products. Part II – Pathogenesis. Ann Agric Environ Med. (2018) 25:186–203. 10.26444/aaem/85651 29575852

[3] Yu H Jing H Chen Z Zheng H Zhu X Wang H. Human *Streptococcus suis* outbreak, Sichuan, China. Emerg Infect Dis. (2006) 12:914–20. 10.3201/eid1206.051194 16707046 PMC3373052

[4] Kerdsin A Dejsirilert S Akeda Y Sekizaki T Hamada S Gottschalk M. Fifteen *Streptococcus suis* serotypes identified by multiplex PCR. J Med Microbiol. (2012) 61:1669–72. 10.1099/jmm.0.048587-0 22918870

[5] Srinivasan V McGee L Njanpop-Lafourcade BM Moisi J Beall B. Species-specific real-time PCR assay for the detection of *Streptococcus suis* from clinical specimens. Diagn Microbiol Infect Dis. (2016) 85:131–2. 10.1016/j.diagmicrobio.2016.02.013 27041105

[6] Zhang J Zhu J Ren H Zhu S Zhao P Zhang F. Rapid visual detection of highly pathogenic Streptococcus *suis* serotype 2 isolates by use of loop-mediated isothermal amplification. J Clin Microbiol. (2013) 51:3250–6. 10.1128/JCM.01183-13 23884995 PMC3811624

[7] Li Y Martinez G Gottschalk M Lacouture S Willson P Dubreuil JD. Identification of a surface protein of *Streptococcus suis* and evaluation of its immunogenic and protective capacity in pigs. Infect Immun. (2006) 74:305–12. 10.1128/IAI.74.1.305-312.2006 16368985 PMC1346615

[8] Feng Y Zheng F Pan X Sun W Wang C Dong Y. Existence and characterization of allelic variants of Sao, a newly identified surface protein from *Streptococcus suis*. FEMS Microbiol Lett. (2007) 275:80–8. 10.1111/j.1574-6968.2007.00859.x 17854470 PMC7110054

[9] Wang L Deng X Liu H Zhao L You X Dai P. The mimic epitopes of *Mycobacterium tuberculosis* screened by phage display peptide library have serodiagnostic potential for tuberculosis. Pathog Dis. (2016) 74:ftw091. 10.1093/femspd/ftw091 27609463

[10] Chandrashekar R Beall MJ Thatcher B Saucier JM Tyrrell P Lappin MR. Serologic responses to peptides of *Anaplasma phagocytophilum* and *Borrelia burgdorferi* in dogs infested with wild-caught *Ixodes scapularis*. Vet J. (2017) 226:6–11. 10.1016/j.tvjl.2017.06.005 28911844

[11] Abdelgawad A Hermes R Damiani A Lamglait B Czirjak GA East M. Comprehensive serology based on a peptide ELISA to assess the prevalence of closely related equine herpesviruses in zoo and wild animals. PLoS ONE. (2015) 10:e138370. 10.1371/journal.pone.0138370 26378452 PMC4574707

[12] Xu GJ Kula T Xu Q Li MZ Vernon SD Ndung U. Viral immunology. Comprehensive serological profiling of human populations using a synthetic human virome. Science. (2015) 348:a698. 10.1126/science.aaa0698 26045439 PMC4844011

[13] van der Wal FJ Jelsma T Fijten H Achterberg RP Loeffen W. Towards a peptide-based suspension array for the detection of pestivirus antibodies in swine. J Virol Methods. (2016) 235:15–20. 10.1016/j.jviromet.2016.04.022 27166561

[14] Lee AJ Bhattacharya R Scheuermann RH Pickett BE. Identification of diagnostic peptide regions that distinguish Zika virus from related mosquito-borne Flaviviruses. PLoS ONE. (2017) 12:e178199. 10.1371/journal.pone.0178199 28562637 PMC5451039

[15] Lu Y Li Z Teng H Xu H Qi S He J. Chimeric peptide constructs comprising linear B-cell epitopes: application to the serodiagnosis of infectious diseases. Sci Rep. (2015) 5:13364. 10.1038/srep13364 26293607 PMC4543967

[16] Feliciano ND Ribeiro VS Gonzaga HT Santos FA Fujimura PT Goulart LR. Short epitope-based synthetic peptides for serodiagnosis of human strongyloidiasis. Immunol Lett. (2016) 172:89–93. 10.1016/j.imlet.2016.03.002 26956434

[17] Carvalho G Resende DM Siqueira L Lopes MD Lopes DO Coelho P. Selecting targets for the diagnosis of *Schistosoma mansoni* infection: an integrative approach using multi-omic and immunoinformatics data. PLoS ONE. (2017) 12:e182299. 10.1371/journal.pone.0182299 28817585 PMC5560627

[18] Okura M Osaki M Nomoto R Arai S Osawa R Sekizaki T. Current taxonomical situation of *Streptococcus suis*. Pathogens. (2016) 5:45. 10.3390/pathogens5030045 27348006 PMC5039425

[19] Estrada AA Gottschalk M Rossow S Rendahl A Gebhart C Marthaler DG. Serotype and genotype (multilocus sequence type) of *Streptococcus suis* isolates from the United States serve as predictors of pathotype. J Clin Microbiol. (2019) 57:e00377–19. 10.1128/JCM.00377-19 31243086 PMC6711919

[20] Goyette-Desjardins G Auger JP Xu J Segura M Gottschalk M. *Streptococcus suis*, an important pig pathogen and emerging zoonotic agent-an update on the worldwide distribution based on serotyping and sequence typing. Emerg Microbes Infect. (2014) 3:e45. 10.1038/emi.2014.45 26038745 PMC4078792

